# Effect of Al and Mo Redistribution on α/β Interface Stability in Dual-Phase Titanium Alloys During Plastic Deformation

**DOI:** 10.3390/ma19112308

**Published:** 2026-05-29

**Authors:** Wenyu Zhang, Mingjie Shi, Ziyu Guo, Shangyi Ma, Qiujie Chen

**Affiliations:** 1 Shenyang National Laboratory for Materials Science, Institute of Metal Research, Chinese Academy of Sciences, Shenyang 110016, China; wyzhang18s@imr.ac.cn; 2 School of Materials Science and Engineering, University of Science and Technology of China, Shenyang 110016, China; 3 Western Metal Materials Co., Ltd., Xi’an 710200, China; smj15617632146@163.com; 4 State Key Laboratory of Digital Steel, Northeastern University, Shenyang 110819, China; guozy8@mails.neu.edu.cn; 5 Ningbo Institute of Technology, Beihang University, Ningbo 315800, China

**Keywords:** dual-phase titanium alloy, mechanical behaviors, α/β interfaces, elemental redistribution, first-principles calculations

## Abstract

The TC11 α + β dual-phase titanium alloy exhibits limited room-temperature ductility (3.3 × 10^−4^ s^−1^: elongation 13.8%) but achieves significant superplasticity at 900 °C (3.3 × 10^−4^ s^−1^: elongation 314%), which correlates strongly with the mechanical response of α/β interfaces. These interfaces, which often crack at room temperature, undergo extensive sliding while preserving structural integrity during superplastic deformation. Combining microstructural analysis with first principles calculations, this study reveals how the stability of the α/β interface is dominated by the redistribution of alloying elements, thereby leading to distinct mechanical behaviors. Energy-dispersive X-ray spectroscopy results and calculated solution energies demonstrate that Mo preferentially dissolves in the β phase, whereas Al exhibits comparable solubility in both phases with a slight preference for the α phase. During high-temperature deformation, the α→β transformation drives Mo redistribution away from the interface toward newly formed β phases. This redistribution of Mo lowers the interfacial energy, strengthens the interface, suppresses stress-induced cracking, and ensures macroscopic continuity. Our study provides a theoretical perspective for Ti alloy design through interfacial engineering.

## 1. Introduction

Superplastic forming of α + β titanium alloys exploits fine-grained two-phase microstructures capable of tensile elongation to several hundred percent at elevated temperatures without macroscopic fracture [1,2,3,4]. Extensive research has established that grain and phase boundary sliding (GBS) is the dominant strain-accommodation mechanism, with α/β interfaces exhibiting the lowest sliding resistance among all boundary types [5,6,7,8]. At room temperature, the same interfaces act as strong barriers to dislocation motion, leading to pile-up, stress concentration, and premature cracking [9,10,11]. The transition from brittle to sliding behavior with increasing temperature was long attributed simplistically to enhanced dislocation mobility; however, it is now recognized that multiple complementary mechanisms operate: GBS is accommodated by interfacial and lattice diffusion [12,13], climb and glide of lattice dislocations that relieve stress at triple junctions [2], and local changes in interfacial chemistry that alter cohesion and diffusivity [14,15].

In α + β Ti alloys, the α ↔ β phase transformation during thermomechanical processing drives marked partitioning of alloying elements, with β-stabilizing elements such as Mo and V enriching in the β phase and α-stabilizers like Al concentrating in the α phase [16,17,18]. Composition gradients are steepest within a few nanometers of the α/β interface. Segregation of slow-diffusing Mo atoms at these boundaries has been observed to reduce interfacial diffusivity, suppress micro-void nucleation, and improve high-temperature tensile strength [19,20,21]. First-principles calculations further reveal that solute redistribution can alter the interfacial electronic density of states and modify the ideal work of separation, with important consequences for interfacial cohesion [22,23]. Nevertheless, these findings have been obtained largely in isolation: experimentally measured composition profiles are rarely combined with interface-specific crystallographic character and with quantum-mechanical bonding analysis. What remains unresolved is how the redistribution of key alloying elements, especially Mo and Al, affects the local stability of α/β interfaces and consequently governs the competition between decohesion and sliding under superplastic conditions. This gap prevents the deliberate tailoring of interfacial chemistry to enhance superplastic formability.

To address this gap, the present study employs a multiscale strategy that integrates scanning electron microscopy (SEM), scanning electron microscopy energy-dispersive X-ray spectroscopy (SEM-EDS), electron backscatter diffraction (EBSD), and density functional theory (DFT) calculations. By correlating nanometer-scale composition variations across individual α/β interfaces with their crystallographic character and computed cohesive energies, this combined approach reveals how phase-transformation-induced redistribution of Mo and Al modifies interfacial electronic structure and directly regulates mechanical stability. The work clarifies the effect of elemental redistribution on α/β interface stability and provides a theoretical basis for interfacial engineering in superplastic α + β titanium alloys.

## 2. Materials and Methods

The α + β dual-phase TC11 Ti alloy with a nominal composition of Ti-6.44Al-3.40Mo-1.66Zr-0.24Si was used in our study. It consists of 47.8% primary α (α_p_) and 52.2% secondary α (α_s_) and β phases. Among the major alloying elements, Al acts as a typical α-phase stabilizer, while Mo serves as a strong β-phase stabilizer, which are the two key elements dominating the phase stability and interfacial behavior of the α + β dual-phase alloy. Zr and Si are important neutral elements and eutectoid β-stabilizing elements that influence the mechanical properties and microstructural stability of the alloy, respectively. Compared to Al and Mo, Zr and Si have a relatively weaker effect on solute distribution and interfacial energetics and are present in lower concentrations.

To systematically investigate phase evolution and elemental redistribution at elevated temperatures, stress-free annealing experiments were conducted. Rectangular samples (20 × 15 × 12 mm^3^) were heated to 850 °C, 900 °C, 950 °C, and 1050 °C in a resistance furnace, held for 30 min to ensure equilibrium, and subsequently water-quenched to freeze the high-temperature microstructure. Then, the billets were machined into tensile test specimens—M8Φ4 (total length: 54 mm; grip ends: M8 × 1.25 threads, 10 mm long; parallel section: 25 mm long, 4 mm in diameter). Tensile tests were performed at room temperature and 900 °C using a Shimadzu AG-X 250 kN universal testing machine (Shimadzu Corporation, Kyoto, Japan) with different strain rates (3.3 × 10^−4^ s^−1^, 0.01 s^−1^ and 0.1 s^−1^), and each condition was repeated 3~4 times to ensure data reliability. The metallographic surfaces were mechanically ground, polished, and etched with Kroll’s reagent (HF:HNO_3_:H_2_O = 2:5:43) for approximately 10 s. Microstructural characterization and fracture surface analysis were conducted using a TESCAN MIRA3 field-emission scanning electron microscope (SEM) (TESCAN Brno, s.r.o., Brno, Czech Republic). The partitioning behavior of Al and Mo within the α_p_, secondary α (α_s_), and β phases was quantitatively analyzed using an Oxford Instruments energy-dispersive X-ray spectrometer (EDS) (TESCAN Brno, s.r.o., Brno, Czech Republic) equipped on the SEM. For EBSD analysis, a specimen of initial material was prepared by mechanical polishing followed by electropolishing in a solution of 90 vol% ethanol and 10 vol% perchloric acid at −40 °C and 30 V for 3 min. The EBSD measurements were conducted using the TESCAN MIRA3 with a scanning step size of 0.17 µm, and data post-processing was performed via HKL Channel 5 software.

**Figure 1:**
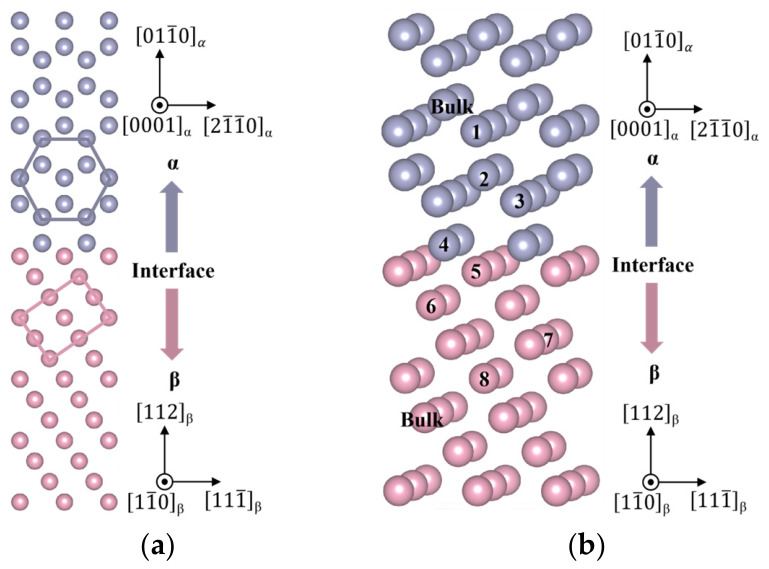
Atomic structure models of the α/β interface in titanium. (**a**) Side view of the interface supercell; (**b**) schematic illustration of solute substitutional sites within the interfacial region (labeled 1–6) and the bulk phase (labeled Bulk).

DFT calculations were performed using VASP with the PBE functional and PAW method. A 450 eV cutoff and 0.15 Å^−1^ k-point spacing ensured convergence. Optimizations continued until forces were below 0.01 eV/Å. An α/β interface model was built based on the Burgers orientation relationship, as shown in Figure 1 [24]. Because unconstrained β-Ti collapses from bcc at 0 K [25,26], the β atoms were restricted to normal relaxation only—a constraint that retains the experimentally relevant bcc structure but neglects in-plane distortions that may modestly influence segregation behavior. α-Ti atoms were fully relaxed in all three directions. Six substitutional sites with different distances to the interface were selected to investigate segregation behaviors of elements.

## 3. Results

### 3.1. Mechanical Behavior and Fracture Observations

**Figure 2:**
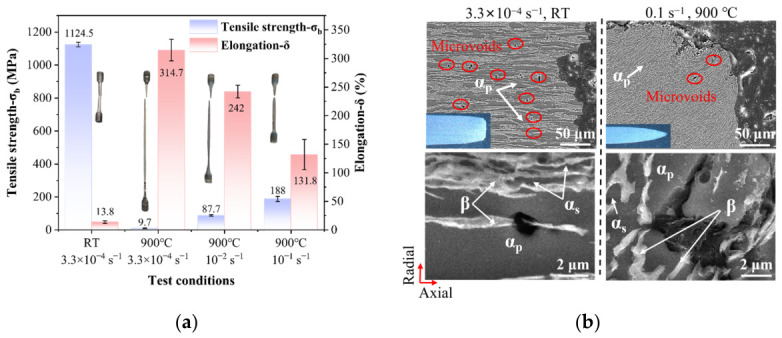
Tensile tests and fracture analysis. (**a**) Mechanical behaviors and post-test specimens under different temperature and strain rates; (**b**) SEM micrographs near the fracture tip.

As shown in Figure 2a, the tensile behaviors of the TC11 alloy depend on temperature and strain rate. Under room-temperature (RT) quasi-static conditions (3.3 × 10^−4^ s^−1^), the alloy displays high tensile strength (σ_b_ = 1124.5 MPa) but limited ductility, with an elongation (δ) of only 13.8%. However, increasing the deformation temperature to 900 °C triggers a dramatic transition: the tensile strength drops sharply to 9.7 MPa, while the elongation surges to 314.7%, manifesting typical superplastic behavior. Even at a higher strain rate of 0.1 s^−1^ at 900 °C, the alloy retains a substantial elongation of 131.8% with a moderate strength of 188 MPa. This high strain-rate sensitivity and thermal softening capability are indicative of a transition in the dominant deformation mechanism, likely involving extensive grain/phase boundary sliding [9].

The microstructural evolution and damage mechanisms accompanying this transition were further characterized by SEM (Figure 2b). At room temperature, the fracture mode is dominated by the nucleation of microvoids, which are preferentially located at the α/β phase boundaries. As indicated by the red circles, these interfacial voids tend to coalesce along the tensile axis, leading to premature macroscopic failure. This preferential nucleation suggests that the α/β interface acts as the weakest link during low-temperature deformation, owing to insufficient interfacial cohesion to accommodate the strain mismatch between the hard α and soft β phases [24]. In stark contrast, specimens deformed at 900 °C exhibit a significantly reduced density of microvoids near the fracture surface. The damage zone is confined to within 50 μm from the fracture tip, compared to over 200 μm at RT. Crucially, despite the large plastic strain (131.8%), the α/β interfaces remain largely intact without severe decohesion. This suppression of interfacial cavitation implies that the cohesive strength and stability of the α/β interface are significantly enhanced at elevated temperatures, facilitating cooperative phase boundary sliding rather than cracking.

### 3.2. Characteristics of Microstructure and Element Distribution

**Figure 3:**
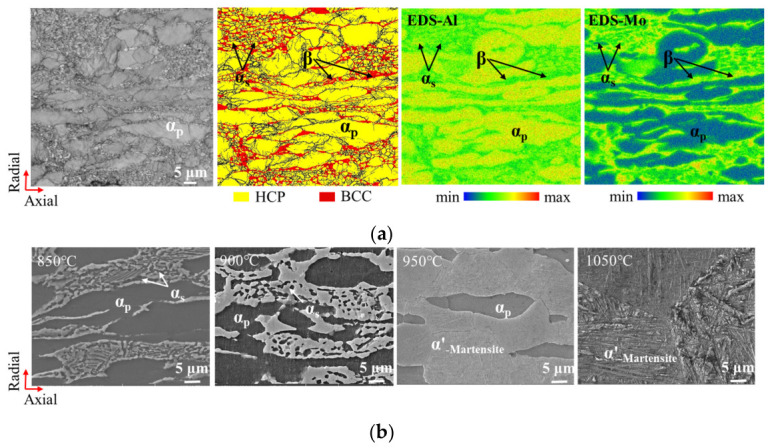
Microstructural analysis of the TC11 alloy. (**a**) EBSD and EDS examinations of the initial microstructure; (**b**) Microstructure at 850 °C, 900 °C, 950 °C, and 1050 °C.

To elucidate the microstructural mechanism of interfacial toughening during tension at 900 °C, the characteristics of α and β phases and distribution of Al and Mo elements in the initial microstructure were analyzed via EBSD and EDS, respectively. As shown in Table 1, quantitative analysis of the distribution of Al and Mo elements in α and β phases was performed via EDS on both initial samples and those after annealing at high temperatures (850–1050 °C). The initial microstructure of the TC11 alloy exhibited coarse and axially elongated primary α phase (α_p_) with an extended α_p_/β interface, whereas the secondary α phase (α_s_) was finer and interlaced with the β phase (Figure 3a). Consistent with the partitioning behavior reported in dual-phase titanium alloys [13], Al acts as an α-stabilizer (Figure 3a), preferentially enriching the α phase (particularly α_p_) and maintaining a stable concentration profile insensitive to temperature. Conversely, Mo, a strong β-stabilizer, exhibits a temperature-dependent redistribution behavior. Notably, a distinct concentration discrepancy is observed between the α phase variants: the Mo content in secondary αs laths (3.81–5.78 wt.%) is significantly higher than that in primary αp grains (<1.01 wt.%). This enrichment arises because α_s_ precipitates from the Mo-rich β matrix during cooling, inevitably inheriting a portion of the solute atoms due to incomplete diffusion kinetics. This observation validates the sensitivity of the EDS measurements in distinguishing the chemical characteristics of different phase morphologies. As the temperature rises to 900 °C, the dissolution of α_s_ phases triggers a significant decrease in Mo concentration within the β matrix (from 8.70 wt.% to 6.34 wt.%), while the Mo concentration in the residual α_s_ phase gradually increases (from 3.81 wt% to 5.62 wt%), indicating a trend toward elemental homogenization. As demonstrated by Du et al. [27], the local concentration of Mo plays a decisive role in governing deformation mechanisms and ductile-to-brittle transitions. Here, the dynamic redistribution is governed by the α to β phase transformation. The dissolution of the α phase at elevated temperatures expands the β matrix volume, naturally diluting the Mo concentration within the β phase (Figure 3b). This compositional homogenization alters the local chemical environment adjacent to the α/β interface. Validating how such variations in local solute concentration govern interfacial cohesion constitutes the physical basis for the atomic-scale analysis discussed in the following sections.

### 3.3. First-Principles Analysis

To quantitatively investigate element redistribution, the solution energies *E_sol_* of Mo and Al in α and β phases, as well as their segregation energies *E_seg_* at α/β interface, were calculated. The solution energy *E_sol_* is defined as:

(1) $$E_{\mathrm{sol}}\;=\;\frac{E_{\mathrm{bulk}}^{\mathrm{Ti}-X}-E_{\mathrm{bulk}}^{\mathrm{Ti}}-mE_{\mathrm{bulk}}^{X}}{m},$$

where $E_{\mathrm{bulk}}^{\mathrm{Ti}-X}$ represents the total energy of a supercell containing (100 − *m*) Ti atoms and m solute X atoms, $E_{\mathrm{bulk}}^{\mathrm{Ti}}$ denotes the total energy of a pure Ti supercell with (100 − *m*) Ti atoms, and $E_{\mathrm{bulk}}^{X}$ is the single-atom energy of solute element X in its stable reference state. A negative *E_sol_* indicates that the dissolution of the solute in the host phase is thermodynamically favorable relative to the elemental reference state, with more negative values implying stronger phase preference.

The segregation energy *E_seg_* is defined as:

(2) $$E_{\mathrm{seg}}=E_{\mathrm{IF}-\mathrm{Site}}^{\mathrm{Ti}-X}-E_{\mathrm{Bulk}-\mathrm{Site}}^{\mathrm{Ti}-X}\;,$$

where $E_{\mathrm{IF}-\mathrm{Site}}^{\mathrm{Ti}-X}$ denotes the total energy of the interface supercell with the solute occupying the interface region (Sites 1–6), and $E_{\mathrm{Bulk}-\mathrm{Site}}^{\mathrm{Ti}-X}$ represents the total energy with the solute located at the bulk-like positions (α-side and β-side), respectively. A negative *E_seg_* indicates that the solute preferentially segregates to the interface site relative to the corresponding bulk-like site, which serves as the reference state; a positive value implies the solute favors remaining in the bulk phase.

**Figure 4:**
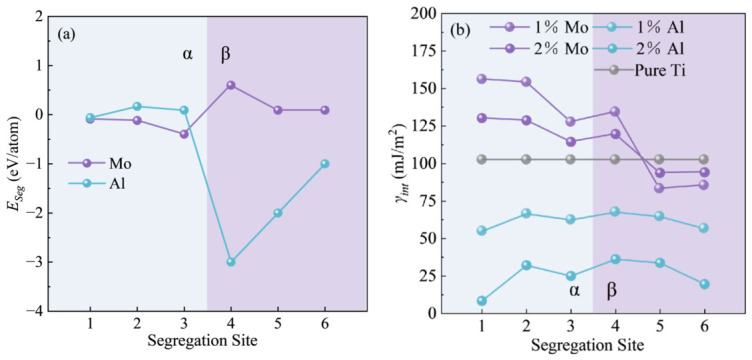
(**a**) Interfacial segregation energy for Al and Mo solutes and (**b**) α/β interfacial energy variations at different Al and Mo doping concentrations.

As listed in Table 2, the *E_sol_* values of Mo in the α and β phases are 0.18 and −0.73 eV, respectively, indicating Mo preferentially dissolves in the β phase, which is consistent with the EDS analysis. In contrast, Al shows comparable *E_sol_* values of −0.99 eV and −0.94 eV in the α and β phases, resulting in a uniform distribution. Thus, in the present calculations, Mo is strongly β-stabilizing, whereas Al exhibits a relatively small phase preference, consistent with the partitioning behavior observed in the EDS analysis. Regarding element segregation, Al predominantly segregates at the β side of the α/β interface rather than at the α side, with the *E_seg_* values of −3.16 eV and ~0 eV, respectively, as shown in Figure 4a. Conversely, Mo tends to segregate at the α side, as indicated by its negative *E_seg_* value. Whereas its positive *E_seg_* at the β side suggests a preference for dissolving in the β bulk over segregating at the interface.

The contributions of element segregation to interfacial stability were evaluated through interfacial energy $\gamma_{\mathrm{int}}$. The interfacial energy $\gamma_{\mathrm{int}}$ is defined as:

(3) $$\gamma_{\mathrm{int}}=\frac{E_{\mathrm{int}}^{\mathrm{Ti}-X}-N_{\alpha }E_{\mathrm{HCP}-\mathrm{Ti}}^{\mathrm{bulk}}-N_{\beta }E_{\mathrm{BCC}-\mathrm{Ti}}^{\mathrm{bulk}}}{A}-\sigma_{\alpha }-\sigma_{\beta },$$

where A is the interfacial area, $E_{\mathrm{int}}^{\mathrm{Ti}-X}$ is the total energy of the doped interface supercell, $N_{\alpha }$ and $N_{\beta }$ denote the number of atoms in α or β phase and $E^{\mathrm{bulk}}$ represent the corresponding single-atom energies of pure Ti in the HCP and BCC structures. $\sigma_{\alpha }$ and $\sigma_{\beta }$ represents the surface energy of the constituent phases.

Though these contributions are site- and phase-dependent, our calculations indicate Al segregation at both sides of the α/β interface reduces interfacial energy and enhances interface stability, particularly at higher concentrations as shown in Figure 4b. Here, the 1% and 2% concentrations refer to one and two solute atoms, respectively, substituting at the same atomic layer within the interfacial supercell, corresponding to approximately 1 at.% and 2 at.% of the total ~100 atoms in the model; these values are chosen to be illustrative of concentration-dependent trends rather than exact replicas of the experimentally measured local compositions. Different from Al, Mo segregation at the α side or β region close to the α phase may weaken the α/β interface. However, Mo segregation at sites 5 and 6 within the β phase, slightly far from the α/β interface, can reduce the interfacial energy, especially at low concentrations. It indicates that redistributing Mo toward the β phase, rather than the α/β interface, can improve interface stability. EDS reveals the concentrations of Mo in the β phase decrease with increasing temperature, which is attributed to the redistribution of Mo toward the newly formed β phase upon the α→β transformation. This reduction in Mo concentrations near the α/β interface actually enhances the interface stability, which may also account for the preserved integrity of the α/β interface and inhibition of crack initiation during superplastic deformation of TC11 at elevated temperature. Consequently, the stability of the α/β interface in the TC11 alloy is highly dominated by the distribution of Al and Mo. The redistribution of Mo induced by α→β transformation reduces the interfacial energy. The reduced interfacial energy strengthens the α/β interface and suppresses cracking induced by stress concentration, thereby ensuring macroscopic continuity during plastic deformation [5,21,28]. It should be noted that the present DFT model considers a simplified Ti–Mo/Al system with an ideal, planar α/β interface, whereas the actual TC11 alloy contains additional alloying elements (e.g., Zr, Si) and exhibits microstructural heterogeneity, including interface curvature, local strain fields, and concurrent elemental interactions. These factors may further modulate segregation behavior and interfacial stability beyond what is captured here; the current calculations therefore provide a mechanistic framework for the dominant effects of Mo and Al redistribution rather than a fully quantitative description of the industrial alloy.

## 4. Discussion

The integrated SEM/EDS/EBSD and DFT results establish a causal link between solute redistribution and α/β interfacial stability in the TC11 alloy. EDS demonstrates that the Mo concentration in the β phase near the α/β interface decreases from approximately 8.70 wt.% in the as-received billet to 6.34 wt.% after high-temperature deformation, whereas the Al partitioning profile remains relatively stable across the interface. DFT calculations reveal that a high Mo content at the β side of the interface increases the interfacial energy and reduces cohesion, while dilution of Mo at β-side bulk-like sites significantly lowers the interfacial energy and enhances stability; Al segregation provides a continuous stabilizing effect largely insensitive to concentration variations. Together, these findings demonstrate that the α→β phase transformation at 900 °C triggers a Mo dilution effect that thermodynamically stabilizes the α/β interface, transforming it from a crack-prone boundary into a strengthened interface capable of withstanding superplastic deformation.

Several aspects of the proposed mechanism remain inferential. The SEM-EDS provides compositional information averaged over an interaction volume of several nanometers, which is insufficient to resolve the atomically sharp Mo depletion profile at the interface plane where DFT predicts the most pronounced energetic effects. The DFT model employs a simplified Ti–Mo/Al binary-like substitutional system with an ideal planar Burgers-oriented interface, whereas the actual TC11 alloy contains additional elements, including Zr, Si, and Fe, that may co-segregate and modify interfacial energetics in ways not captured here. The causal relationship between the predicted reduction in interfacial energy and the observed suppression of interfacial cracking is inferred from post-mortem observations without direct in situ evidence. The limited number of mechanical test specimens constrains the statistical robustness of the correlation between Mo concentration and crack density.

At 900 °C, approximately 0.6 of the absolute melting temperature for the TC11 alloy, the Mo dilution mechanism does not operate in isolation. Diffusional accommodation, including both interfacial diffusion and lattice self-diffusion, can effectively relax stress concentrations that accumulate at interface triple junctions during grain boundary sliding, preventing local tensile stresses from exceeding the interfacial cohesive strength and nucleating microvoids. A thermodynamically more stable interface may further enhance this relaxation by presenting lower energy barriers for atomic migration. Additionally, the semicoherent Burgers-oriented α/β interface undergoes dynamic structural adjustment during deformation, including misfit dislocation climb and interfacial ledge rearrangement, which redistribute local stress and eliminate potential decohesion sites. Extensive grain boundary sliding itself may promote further solute redistribution by generating excess vacancies and enhancing pipe diffusion along interfacial dislocations, creating a positive feedback loop between mechanical deformation and chemical stabilization.

The DFT model employs a simplified Ti–Mo/Al system and does not account for the multicomponent nature of the TC11 alloy nor for microstructural features such as interface curvature and local strain fields. The interfacial cracking analysis relies on post-mortem observations rather than in situ tracking of crack initiation. The DFT calculations are performed at 0 K with constrained β-Ti relaxation, and while this preserves the experimentally relevant bcc structure, finite-temperature contributions such as vibrational entropy are not included. These limitations indicate that the present work provides a mechanistic framework rather than a fully quantitative description.

The finding that Mo redistribution governs interfacial stability carries practical implications for alloy processing and design. Pre-deformation heat treatments that pre-dilute Mo near interfaces through step-annealing in the α + β phase field followed by controlled cooling could precondition the interfacial state for enhanced stability during subsequent superplastic forming. Multi-stage deformation schedules alternating between α + β and β phase field processing may promote progressive Mo homogenization away from interfaces, cumulatively improving interfacial cohesion. The cooling rate from the hot deformation temperature directly influences the extent of Mo partitioning during the reverse β→α transformation, and rapid cooling may suppress detrimental Mo enrichment at newly formed interfaces. Minor additions of slow-diffusing β-stabilizing elements beyond Mo, or trace elements that compete for interfacial segregation sites, could be explored to further modulate interfacial chemistry. More broadly, this study suggests that interfacial engineering, defined as the deliberate manipulation of local phase-boundary chemistry through thermal and mechanical processing, represents a viable strategy for optimizing the superplastic formability of α + β titanium alloys, complementing established approaches of grain refinement and phase proportion control.

## 5. Conclusions

This study clarifies how the redistribution of Al and Mo governs α/β interfacial stability in TC11 titanium alloy during superplastic forming. EDS analysis reveals that Mo preferentially partitions into the β phase, with its concentration near the α/β interface decreasing from ~8.70 wt.% to ~6.34 wt.% after high-temperature deformation, while Al distributes nearly uniformly across both phases with a slight α preference. DFT calculations demonstrate that Al segregation at either side of the α/β interface consistently reduces interfacial energy and enhances stability, whereas Mo segregation at the α side or the β region immediately adjacent to the interface increases interfacial energy and weakens cohesion. The α→β phase transformation at elevated temperature drives Mo away from the interface into the newly formed β phase, thereby diluting the interfacial Mo concentration and thermodynamically stabilizing the interface. Microstructural observations show that this enhanced interfacial stability correlates with suppressed interfacial cracking and preserved microstructural integrity during superplastic deformation, a relationship inferred from post-mortem analysis rather than direct in situ interfacial strength measurements. These findings suggest that controlling Mo redistribution through thermal and mechanical processing offers a practical route for interfacial engineering to improve the superplastic formability of α + β titanium alloys.

## Figures and Tables

**Table 1:** EDS analysis of the TC11 alloy subjected to different annealing temperatures. (“-” indicates that the corresponding phase dissolves at this temperature).

Solute	Phase	Billet	850 °C	900 °C	950 °C	1050 °C
Mo (wt.%)	α_p_	0.66 ± 0.10	0.74 ± 0.20	0.72 ± 0.12	0.78 ± 0.43	-
	α_s_	3.81 ± 0.58	5.78 ± 1.65	5.62 ± 0.68	-	-
	β	8.70 ± 0.61	6.59 ± 1.23	6.34 ± 1.31	4.73 ± 0.43	3.67 ± 0.25
Al (wt.%)	α_p_	7.11 ± 0.19	6.79 ± 0.12	7.02 ± 0.07	6.98 ± 0.19	-
	α_s_	4.95 ± 0.09	4.63 ± 0.31	5.04 ± 0.09	-	-
	β	5.85 ± 0.59	5.86 ± 0.78	5.38 ± 0.32	5.69 ± 0.24	5.95 ± 0.17

**Table 2:** Calculated solution energies (eV/atom) of Al and Mo atoms in α- and β-Ti phases.

Solute	Mo		Al	
Phase	α	β	α	β
Solution energy	0.18	−0.73	−0.99	−0.94

## Data Availability

The original contributions presented in this study are included in the article. Further inquiries can be directed to the corresponding authors.
